# Subperiosteal Resection

**Published:** 1869-01

**Authors:** 


Subperiosteal Resection.—Before the Medical Society of Lyons, M. Ollier recently presented four patients upon whom had been practiced subperiosteal resection of the elbow joint. The first was a young girl thirteen years old, and the operation dated from ninety days. Resection had been made of all the inferior epiphysis of the humerus, to the extent of thirty millimetres, and of the superior extremities of the cubitus and radius. There had been reproduction of bone and complete cicatrization; the movements of flexion were entirely re-established, with the power of semi-extension and semi supination.

The second operation dated from fifteen months; resection of four centimetres of the humerus; reproduction of the bone; the patient had been able to resume her duties as cook, and in spite of the occurrence of two attacks of arthritis, she could now execute extended movements of extension and supination.

A third patient, twenty-four years old, some months after resection of the articulation, presents complete cicatrization, perfect reproduction of the bones and movements of flexion and extension, pronation and supination, nearly as extended as in the normal conditions.

The fourth had only just laid aside the plaster bandage, and the muscles were weak from inaction; nevertheless, it was evident that the bone had been reproduced, and movements could be executed to about the third of the normal extent.

In these operations, M. Ollier preceded the resections by carefully detaching the periosteum with his instrument, specially adapted to the purpose. On account of the age of the patients, it could not be expected that this membrane would regenerate osseous tissue to repair the loss of substance of the limb; but it was able to regenerate fibrous and osseous masses, which, accumulating around the articulation, rendered it solid and prevented lateral mobility. This solidity was also insured by the employment of an immovable plaster bandage during all the period of periosteal generation.

It is noticeable that the experiments and clinical facts of Ollier by no means sustain the old theory which regards the periosteum itself as the generator of bone by a process of secretion. In all cases a subperiosteal layer of medullary cells is preserved with the membrane itself, and the osseous tissue developed at their expense, as in the normal process of evolution. If this layer be not preserved, no bone is reproduced.

